# Supplementary material for: Generalised Hodgkin–Huxley model captures human P2X and AMPA receptor currents
Source: J Physiol. 2025 Nov 11;604(1):214–46. doi: 10.1113/JP288880 (PMC12783981; doi:10.1113/JP288880)
Supplement: Supplementary file 2 — Supporting Information S1 Text. It provides optimised biochemical rates and sensitivity analysis of the gHH model, discussion on model feedback, contribution of model components, and recovery time of hP2X1 and hP2X2 receptors. S2 Data. All digitised datasets extracted from human experiments referenced in this article are fully described and documented at https://github.com/poshtkohi/gHH/tree/main/hP2XR-hGluAR-model/data. The public repository includes a README file that specifies the units and receptor subtypes with the relevant experimental conditions. S3 Code. All MATLAB source code of the gHH model, including parameter fitting and simulations, can be found on the GitHub page at https://github.com/poshtkohi/gHH. It comes with a README file that explains the source code hierarchy. [file TJP-604-214-s001.docx]

**Generalised Hodgkin-Huxley Model Captures Human P2X and AMPA Receptor Currents**

**Alireza Poshtkohi^1*^, Brian D. Gulbransen^2^**

^1^School of Physics, Engineering and Computer Science, University of Hertfordshire, Hatfield, Hertfordshire, United Kingdom

^2^Department of Physiology, Michigan State University, Michigan, United States of America

**Email addresses:** [a.poshtkohi@herts.ac.uk](mailto:a.poshtkohi@herts.ac.uk) and [gulbrans@msu.edu](mailto:gulbrans@msu.edu)

# S1. 1. Fitted Biochemical Rates and their Sensitivity Analysis

This document presents the model parameters optimally fitted to the current response of human P2X and AMPA receptors along with their sensitivity analysis (SA) graphs, as influenced by varying concentrations of the agonists ATP and glutamate.

**Human P2X_1_ model**

In this supplementary section, we detail the quantitative adjustments to the kinetic parameters of the hP2X_1_ receptor model, reflecting its response under varied ATP concentrations, as delineated in Tables S1.1 and S1. 2. The model SA comes in Fig. S1. 1.

**Table S1. 1**: This table presents the model-fitted rate constants for the activation and deactivation processes of the hP2X_1_ receptors across different ATP concentrations. The parameters $\alpha_{m_{1}}$, $\beta_{m_{1}}$, $\alpha_{m_{2}}$, and $\beta_{m_{2}}$correspond to the rates of activation and deactivation for the two gating mechanisms, respectively, while $\alpha_{h_{1}}$, $\beta_{h_{1}}$, $\alpha_{h_{2}}$, and $\beta_{h_{2}}$represent the rates for the corresponding inactivation processes. Each set of rate constants is specific to a particular ATP concentration, illustrating the receptor's sensitivity and response to varying levels of ligand presence.

| ATP (µM) | $\boldsymbol{\alpha}_{\boldsymbol{m}_{\boldsymbol{1}}}$ | $\boldsymbol{\beta}_{\boldsymbol{m}_{\boldsymbol{1}}}$ | $\boldsymbol{\alpha}_{\boldsymbol{m}_{\boldsymbol{2}}}$ | $\boldsymbol{\beta}_{\boldsymbol{m}_{\boldsymbol{2}}}$ | $\boldsymbol{\alpha}_{\boldsymbol{h}_{\boldsymbol{1}}}$ | $\boldsymbol{\beta}_{\boldsymbol{h}_{\boldsymbol{1}}}$ | $\boldsymbol{\alpha}_{\boldsymbol{h}_{\boldsymbol{2}}}$ | $\boldsymbol{\beta}_{\boldsymbol{h}_{\boldsymbol{2}}}$ |
| --- | --- | --- | --- | --- | --- | --- | --- | --- |
| 0.1 | 108.089 | 1.887 | 0.249 | 0.511 | 0.251 | 0.420 | 1.051 | 39.419 |
| 0.3 | 35.861 | 1.177 | 0.437 | 1.858 | 0.102 | 0.728 | 6.527 | 12.813 |
| 1 | 10.767 | 1.147 | 1.883 | 4.097 | 0.296 | 0.808 | 0.332 | 2.964 |
| 3 | 8.681 | 1.847 | 3.151 | 1.065 | 0.278 | 3.812 | 0.190 | 0.985 |
| 100 | 4.280 | 5.308 | 13.760 | 0.118 | 5.613 | 33.174 | 0.194 | 0.811 |

**Table S1. 2**: This table delineates the units for each parameter listed in Table S1.1. The rate constants $\alpha_{m_{1}}$ is expressed in µM^-1^s^-1^ that reflects the interaction between ATP concentration and time, highlighting its dependence on ligand concentration. Conversely, $\alpha_{m_{2}}$,
$\beta_{m_{1}}$ and $\beta_{m_{2}}$, along with $\alpha_{h_{1}}$, $\beta_{h_{1}}$, $\alpha_{h_{2}}$, and $\beta_{h_{2}}$are measured in s^-1^, indicating their time-dependent nature independent of ligand concentration.

|  | $\boldsymbol{\alpha}_{\boldsymbol{m}_{\boldsymbol{1}}}$ | $\boldsymbol{\beta}_{\boldsymbol{m}_{\boldsymbol{1}}}$ | $\boldsymbol{\alpha}_{\boldsymbol{m}_{\boldsymbol{2}}}$ | $\boldsymbol{\beta}_{\boldsymbol{m}_{\boldsymbol{2}}}$ | $\boldsymbol{\alpha}_{\boldsymbol{h}_{\boldsymbol{1}}}$ | $\boldsymbol{\beta}_{\boldsymbol{h}_{\boldsymbol{1}}}$ | $\boldsymbol{\alpha}_{\boldsymbol{h}_{\boldsymbol{2}}}$ | $\boldsymbol{\beta}_{\boldsymbol{h}_{\boldsymbol{2}}}$ |
| --- | --- | --- | --- | --- | --- | --- | --- | --- |
| Unit | µM^-1^s^-1^ | s^-1^ | s^-1^ | s^-1^ | s^-1^ | s^-1^ | s^-1^ | s^-1^ |

| 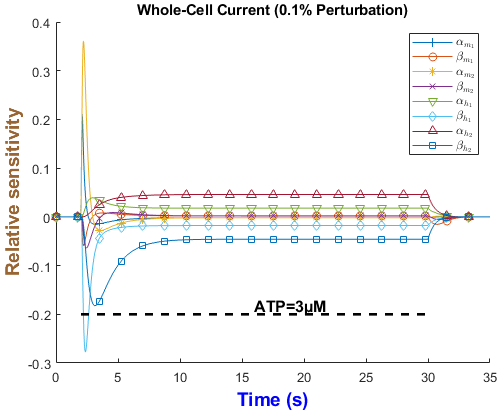  **(A)** | 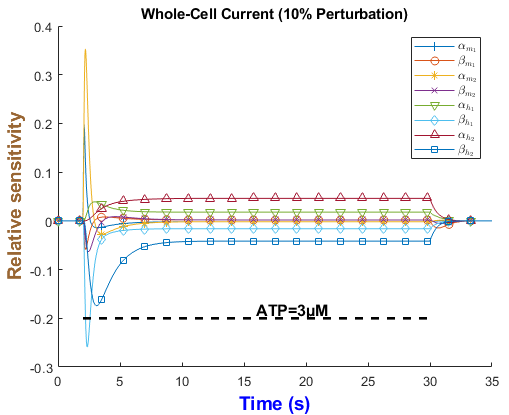  **(B)** |
| --- | --- |

**Figure S1. 1**: Sensitivity analysis of the whole-cell current for ATP=3µM with respect to the parameter set of hP2X_1_ receptor. 0.1% (panel A) and 10% (panel B) perturbation of rate constants.

**Human P2X_2_ model**

This section elaborates on the fitting of the kinetic parameters for the hP2X_2_ receptor model in response to varying ATP concentrations, detailed in Tables S1. 3 and S1.4. The model SA appears in Fig. S1. 2.

**Table S1. 3**: This table delineates the rate constants derived from model fittings for both activation and deactivation phases of the hP2X_2_ receptors, tailored to different ATP concentration scenarios.

| ATP (µM) | $\boldsymbol{\alpha}_{\boldsymbol{m}_{\boldsymbol{1}}}$ | $\boldsymbol{\beta}_{\boldsymbol{m}_{\boldsymbol{1}}}$ | $\boldsymbol{\alpha}_{\boldsymbol{m}_{\boldsymbol{2}}}$ | $\boldsymbol{\beta}_{\boldsymbol{m}_{\boldsymbol{2}}}$ | $\boldsymbol{\alpha}_{\boldsymbol{h}_{\boldsymbol{1}}}$ | $\boldsymbol{\beta}_{\boldsymbol{h}_{\boldsymbol{1}}}$ | $\boldsymbol{\alpha}_{\boldsymbol{h}_{\boldsymbol{2}}}$ | $\boldsymbol{\beta}_{\boldsymbol{h}_{\boldsymbol{2}}}$ |
| --- | --- | --- | --- | --- | --- | --- | --- | --- |
| 3 | 0.0506 | 2.7298 | 5.2221 | 4.3517 | 1.7467 | 400.005 | 1.0172 | 62.0346 |
| 10 | 0.1861 | 1.6117 | 0.0792 | 0.5604 | 7.1185 | 5.5026 | 1.5589 | 99.4609 |
| 30 | 0.1420 | 1.2484 | 0.1809 | 0.4817 | 21.2409 | 6.1716 | 0.9140 | 8.3923 |
| 100 | 0.1218 | 0.7820 | 0.3109 | 0.3844 | 19.6191 | 3.2668 | 0.9182 | 6.0313 |
| 300 | 0.0400 | 0.4702 | 0.1792 | 0.2133 | 27.0249 | 2.6097 | 1.8269 | 14.6965 |

**Table S1. 4**: Units for each parameter listed in Table S1. 3.

|  | $\boldsymbol{\alpha}_{\boldsymbol{m}_{\boldsymbol{1}}}$ | $\boldsymbol{\beta}_{\boldsymbol{m}_{\boldsymbol{1}}}$ | $\boldsymbol{\alpha}_{\boldsymbol{m}_{\boldsymbol{2}}}$ | $\boldsymbol{\beta}_{\boldsymbol{m}_{\boldsymbol{2}}}$ | $\boldsymbol{\alpha}_{\boldsymbol{h}_{\boldsymbol{1}}}$ | $\boldsymbol{\beta}_{\boldsymbol{h}_{\boldsymbol{1}}}$ | $\boldsymbol{\alpha}_{\boldsymbol{h}_{\boldsymbol{2}}}$ | $\boldsymbol{\beta}_{\boldsymbol{h}_{\boldsymbol{2}}}$ |
| --- | --- | --- | --- | --- | --- | --- | --- | --- |
| Unit | µM^-1^s^-1^ | s^-1^ | s^-1^ | s^-1^ | s^-1^ | s^-1^ | s^-1^ | s^-1^ |

| 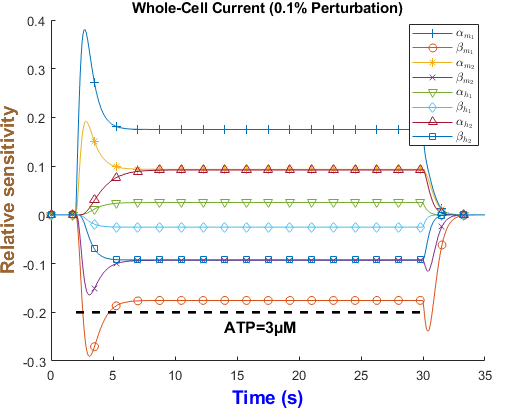  **(A)** | 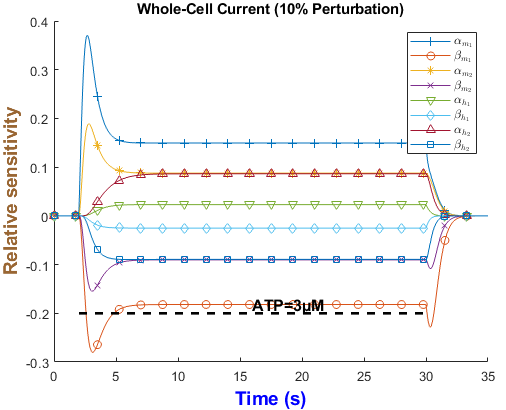  **(B)** |
| --- | --- |

**Figure S1. 2**: Sensitivity analysis of the whole-cell current for ATP=3µM with respect to the parameter set of hP2X_2_ receptor.

**Human P2X_3_ model**

This section presents the calibration of kinetic parameters for the hP2X₃ receptor model across varying ATP concentrations, as detailed in Tables S1. 5 and S1.6. The model parameters were fitted to experimental data, capturing the receptor's activation, inactivation, and desensitisation dynamics under different ATP conditions. The model SA is shown in Fig. S1. 3.

**Table S1. 5**: This table provides the rate constants obtained from model fittings for both activation and inactivation phases of hP2X₃ receptors across a range of ATP concentrationS1. The values illustrate how binding and gating kinetics change in response to increasing ATP levels.

| ATP (µM) | $\boldsymbol{\alpha}_{\boldsymbol{m}_{\boldsymbol{1}}}$ | $\boldsymbol{\beta}_{\boldsymbol{m}_{\boldsymbol{1}}}$ | $\boldsymbol{\alpha}_{\boldsymbol{m}_{\boldsymbol{2}}}$ | $\boldsymbol{\beta}_{\boldsymbol{m}_{\boldsymbol{2}}}$ | $\boldsymbol{\alpha}_{\boldsymbol{h}_{\boldsymbol{1}}}$ | $\boldsymbol{\beta}_{\boldsymbol{h}_{\boldsymbol{1}}}$ | $\boldsymbol{\alpha}_{\boldsymbol{h}_{\boldsymbol{2}}}$ | $\boldsymbol{\beta}_{\boldsymbol{h}_{\boldsymbol{2}}}$ |
| --- | --- | --- | --- | --- | --- | --- | --- | --- |
| 0.1 | 133.257 | 3.5042 | 1.8555 | 2.5567 | 0.9191 | 16.7814 | 0.0898 | 0.2126 |
| 0.3 | 170.521 | 3.4771 | 0.1077 | 1.8879 | 2.3923 | 0.6959 | 0.2216 | 7.3464 |
| 1 | 85.827 | 1.4373 | 0.5289 | 1.3263 | 0.0696 | 3.8155 | 0.0299 | 1.5233 |
| 3 | 14.850 | 0.1292 | 3.3311 | 83.826 | 0.1948 | 0.9096 | 0.1606 | 24.5213 |
| 100 | 4.7519 | 2.1588 | 35.1308 | 110.004 | 0.5134 | 61.941 | 2.4489 | 13.6197 |

**Table S1. 6**: Units for each parameter listed in Table S1. 5.

|  | $\boldsymbol{\alpha}_{\boldsymbol{m}_{\boldsymbol{1}}}$ | $\boldsymbol{\beta}_{\boldsymbol{m}_{\boldsymbol{1}}}$ | $\boldsymbol{\alpha}_{\boldsymbol{m}_{\boldsymbol{2}}}$ | $\boldsymbol{\beta}_{\boldsymbol{m}_{\boldsymbol{2}}}$ | $\boldsymbol{\alpha}_{\boldsymbol{h}_{\boldsymbol{1}}}$ | $\boldsymbol{\beta}_{\boldsymbol{h}_{\boldsymbol{1}}}$ | $\boldsymbol{\alpha}_{\boldsymbol{h}_{\boldsymbol{2}}}$ | $\boldsymbol{\beta}_{\boldsymbol{h}_{\boldsymbol{2}}}$ |
| --- | --- | --- | --- | --- | --- | --- | --- | --- |
| Unit | µM^-1^s^-1^ | s^-1^ | s^-1^ | s^-1^ | s^-1^ | s^-1^ | s^-1^ | s^-1^ |

| 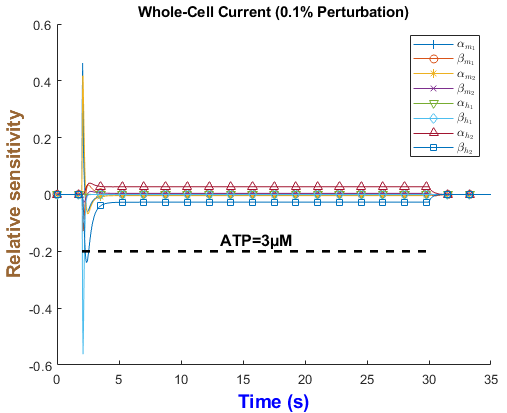  **(A)** | 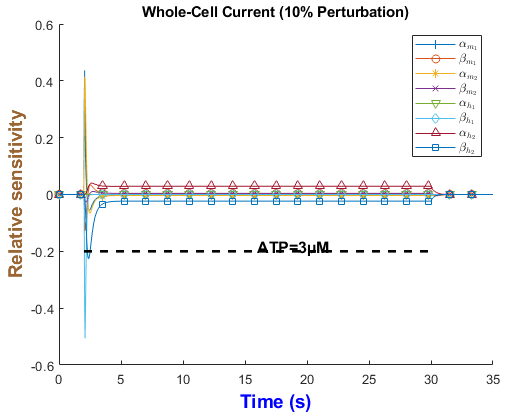  **(B)** |
| --- | --- |

**Figure S1. 3**: Sensitivity analysis of the whole-cell current for ATP=3µM with respect to the parameter set of hP2X_3_ receptor.

**Human P2X_4_ model**

This section details the calibration of kinetic parameters for the hP2X_4_ receptor model across different ATP concentrations, as specified in Tables S1.7 and S1. 8.

**Table S1. 7**: This table presents the rate constants for the activation and deactivation phases of the hP2X_4_ receptors across various ATP concentrations.

| ATP (µM) | $\boldsymbol{\alpha}_{\boldsymbol{m}_{\boldsymbol{1}}}$ | $\boldsymbol{\beta}_{\boldsymbol{m}_{\boldsymbol{1}}}$ | $\boldsymbol{\alpha}_{\boldsymbol{m}_{\boldsymbol{2}}}$ | $\boldsymbol{\beta}_{\boldsymbol{m}_{\boldsymbol{2}}}$ | $\boldsymbol{\alpha}_{\boldsymbol{h}_{\boldsymbol{1}}}$ | $\boldsymbol{\beta}_{\boldsymbol{h}_{\boldsymbol{1}}}$ | $\boldsymbol{\alpha}_{\boldsymbol{h}_{\boldsymbol{2}}}$ | | $\boldsymbol{\beta}_{\boldsymbol{h}_{\boldsymbol{2}}}$ |
| --- | --- | --- | --- | --- | --- | --- | --- | --- | --- |
| 0.5 | 1.8776 | 0.3156 | 0.5910 | 0.4891 | 0.1205 | 2.9297 | 4.1788 | 4.4593 | |
| 1 | 0.8387 | 0.2574 | 0.2315 | 0.3192 | 1.1658 | 3.0766 | 0.0088 | 0.1966 | |
| 5 | 0.3763 | 0.1136 | 2.5514 | 1.8695 | 0.0140 | 2.1342 | 0.0709 | 0.0928 | |
| 10 | 0.9008 | 0.2242 | 0.7775 | 0.0815 | 0.4145 | 75.2454 | 0.2511 | 0.0984 | |
| 50 | 0.0789 | 0.0877 | 0.4407 | 0.5950 | 1.9688 | 2.4333 | 0.0730 | 0.2135 | |
| 100 | 0.0344 | 0.0310 | 0.3946 | 0.4490 | 1.3457 | 1.9245 | 0.0550 | 0.1609 | |
| 500 | 0.0045 | 0.0461 | 0.8242 | 1.5636 | 0.4500 | 0.6468 | 0.0155 | 0.0847 | |

**Table S1. 8**: Units for parameters in Table S1. 7.

|  | $\boldsymbol{\alpha}_{\boldsymbol{m}_{\boldsymbol{1}}}$ | $\boldsymbol{\beta}_{\boldsymbol{m}_{\boldsymbol{1}}}$ | $\boldsymbol{\alpha}_{\boldsymbol{m}_{\boldsymbol{2}}}$ | $\boldsymbol{\beta}_{\boldsymbol{m}_{\boldsymbol{2}}}$ | $\boldsymbol{\alpha}_{\boldsymbol{h}_{\boldsymbol{1}}}$ | $\boldsymbol{\beta}_{\boldsymbol{h}_{\boldsymbol{1}}}$ | $\boldsymbol{\alpha}_{\boldsymbol{h}_{\boldsymbol{2}}}$ | $\boldsymbol{\beta}_{\boldsymbol{h}_{\boldsymbol{2}}}$ |
| --- | --- | --- | --- | --- | --- | --- | --- | --- |
| Unit | µM^-1^s^-1^ | s^-1^ | s^-1^ | s^-1^ | s^-1^ | s^-1^ | s^-1^ | s^-1^ |

**Human P2X_5_ model**

This section outlines the adjustment of kinetic parameters for the hP2X_5_ receptor model in response to varying ATP concentrations, as documented in Tables S1.9 and S1.10. The model SA is demonstrated in Fig. S1. 4.

**Table S1. 9**: This table presents the rate constants for the activation and deactivation phases of the hP2X_5_ receptors across various ATP concentration.

| ATP (µM) | $\boldsymbol{\alpha}_{\boldsymbol{m}_{\boldsymbol{1}}}$ | $\boldsymbol{\beta}_{\boldsymbol{m}_{\boldsymbol{1}}}$ | $\boldsymbol{\alpha}_{\boldsymbol{m}_{\boldsymbol{2}}}$ | $\boldsymbol{\beta}_{\boldsymbol{m}_{\boldsymbol{2}}}$ | $\boldsymbol{\alpha}_{\boldsymbol{h}_{\boldsymbol{1}}}$ | $\boldsymbol{\beta}_{\boldsymbol{h}_{\boldsymbol{1}}}$ | $\boldsymbol{\alpha}_{\boldsymbol{h}_{\boldsymbol{2}}}$ | $\boldsymbol{\beta}_{\boldsymbol{h}_{\boldsymbol{2}}}$ |
| --- | --- | --- | --- | --- | --- | --- | --- | --- |
| 1 | 1.2507 | 0.7277 | 0.1152 | 0.1110 | 10.7844 | 19.2808 | 0.0251 | 1.5159 |
| 3 | 0.6500 | 0.9800 | 0.3379 | 0.0718 | 12.0384 | 6.1044 | 0.0251 | 0.8368 |
| 10 | 1.0259 | 0.1349 | 0.5057 | 1.4617 | 21.3811 | 8.4529 | 0.0331 | 0.1317 |
| 30 | 0.3147 | 0.1606 | 5.3003 | 3.2311 | 27.3618 | 18.2691 | 0.0261 | 0.0132 |
| 100 | 0.0296 | 0.2139 | 1.4458 | 0.5684 | 16.4790 | 9.3359 | 0.1331 | 0.0928 |

**Table S1. 10**: Units for parameters in Table S1. 9.

|  | $\boldsymbol{\alpha}_{\boldsymbol{m}_{\boldsymbol{1}}}$ | $\boldsymbol{\beta}_{\boldsymbol{m}_{\boldsymbol{1}}}$ | $\boldsymbol{\alpha}_{\boldsymbol{m}_{\boldsymbol{2}}}$ | $\boldsymbol{\beta}_{\boldsymbol{m}_{\boldsymbol{2}}}$ | $\boldsymbol{\alpha}_{\boldsymbol{h}_{\boldsymbol{1}}}$ | $\boldsymbol{\beta}_{\boldsymbol{h}_{\boldsymbol{1}}}$ | $\boldsymbol{\alpha}_{\boldsymbol{h}_{\boldsymbol{2}}}$ | $\boldsymbol{\beta}_{\boldsymbol{h}_{\boldsymbol{2}}}$ |
| --- | --- | --- | --- | --- | --- | --- | --- | --- |
| Unit | µM^-1^s^-1^ | s^-1^ | s^-1^ | s^-1^ | s^-1^ | s^-1^ | s^-1^ | s^-1^ |

| 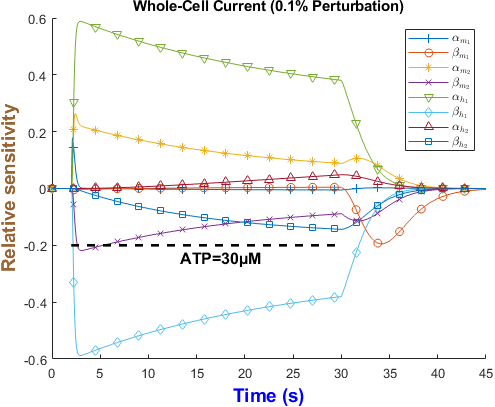  **(A)** | 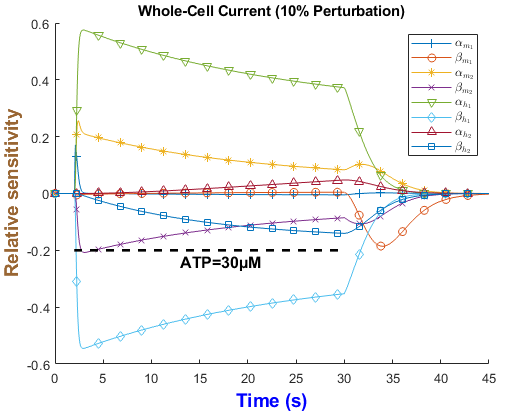  **(B)** |
| --- | --- |

**Figure S1. 4**: Sensitivity analysis of the whole-cell current for ATP=30µM with respect to the parameter set of hP2X_5_ receptor.

**Human P2X_6_ model**

This section details the calibration of kinetic parameters for the hP2X_6_ receptor model, adjusting for variations in ATP concentration. The refined parameters are listed in Tables S1. 11 and S1.12. The model SA appears in Fig. S1. 5.

**Table S1. 11**: Rate constants for activation and deactivation processes of the human P2X_6_ receptor.

| ATP (µM) | $\boldsymbol{\alpha}_{\boldsymbol{m}_{\boldsymbol{1}}}$ | $\boldsymbol{\beta}_{\boldsymbol{m}_{\boldsymbol{1}}}$ | $\boldsymbol{\alpha}_{\boldsymbol{m}_{\boldsymbol{2}}}$ | $\boldsymbol{\beta}_{\boldsymbol{m}_{\boldsymbol{2}}}$ | $\boldsymbol{\alpha}_{\boldsymbol{h}_{\boldsymbol{1}}}$ | $\boldsymbol{\beta}_{\boldsymbol{h}_{\boldsymbol{1}}}$ | $\boldsymbol{\alpha}_{\boldsymbol{h}_{\boldsymbol{2}}}$ | $\boldsymbol{\beta}_{\boldsymbol{h}_{\boldsymbol{2}}}$ |
| --- | --- | --- | --- | --- | --- | --- | --- | --- |
| 30 | 1.5041 | 0.5801 | 0.9157 | 1.2413 | 0.0856 | 0.5795 | 25.8780 | 0.0792 |

**Table S1. 12**: Units of parameters for kinetic modelling detailed in Table S1. 11.

|  | $\boldsymbol{\alpha}_{\boldsymbol{m}_{\boldsymbol{1}}}$ | $\boldsymbol{\beta}_{\boldsymbol{m}_{\boldsymbol{1}}}$ | $\boldsymbol{\alpha}_{\boldsymbol{m}_{\boldsymbol{2}}}$ | $\boldsymbol{\beta}_{\boldsymbol{m}_{\boldsymbol{2}}}$ | $\boldsymbol{\alpha}_{\boldsymbol{h}_{\boldsymbol{1}}}$ | $\boldsymbol{\beta}_{\boldsymbol{h}_{\boldsymbol{1}}}$ | $\boldsymbol{\alpha}_{\boldsymbol{h}_{\boldsymbol{2}}}$ | $\boldsymbol{\beta}_{\boldsymbol{h}_{\boldsymbol{2}}}$ |
| --- | --- | --- | --- | --- | --- | --- | --- | --- |
| Unit | µM^-1^s^-1^ | s^-1^ | s^-1^ | s^-1^ | s^-1^ | s^-1^ | s^-1^ | s^-1^ |

| 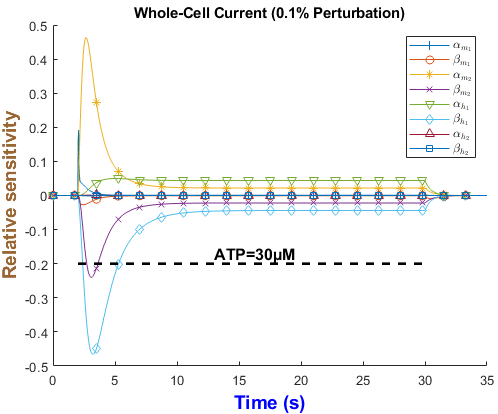  **(A)** | 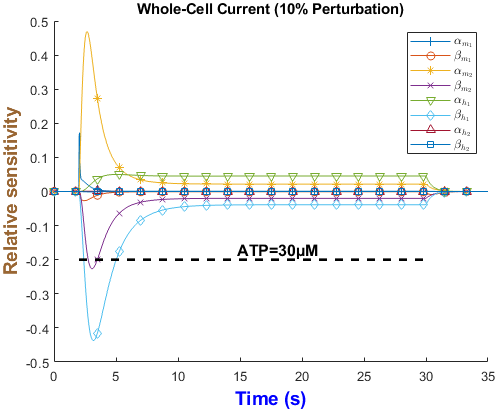  **(B)** |
| --- | --- |

**Figure S1. 5**: Sensitivity analysis of the whole-cell current for ATP=30µM with respect to the parameter set of hP2X_6_ receptor.

**Human P2X_7_ model**

This section presents the refinement of kinetic parameters for the hP2X_7_ receptor model across varying levels of ATP, detailed in Tables S1. 12 and S1. 13. The model SA is depicted in Fig. S1. 6.

**Table S1. 12**: This table displays the rate constants for both activation and deactivation processes of the hP2X_7_ receptors at different ATP concentrations.

| ATP (mM) | $\boldsymbol{\alpha}_{\boldsymbol{m}_{\boldsymbol{1}}}$ | $\boldsymbol{\beta}_{\boldsymbol{m}_{\boldsymbol{1}}}$ | $\boldsymbol{\alpha}_{\boldsymbol{m}_{\boldsymbol{2}}}$ | $\boldsymbol{\beta}_{\boldsymbol{m}_{\boldsymbol{2}}}$ | $\boldsymbol{\alpha}_{\boldsymbol{h}_{\boldsymbol{1}}}$ | $\boldsymbol{\beta}_{\boldsymbol{h}_{\boldsymbol{1}}}$ | $\boldsymbol{\alpha}_{\boldsymbol{h}_{\boldsymbol{2}}}$ | $\boldsymbol{\beta}_{\boldsymbol{h}_{\boldsymbol{2}}}$ |
| --- | --- | --- | --- | --- | --- | --- | --- | --- |
| 0.01 | 128.673 | 0.1435 | 0.0088 | 0.0938 | 7.8275 | 451.12 | 0.1445 | 0.3852 |
| 0.1 | 22.122 | 0.5401 | 0.0392 | 0.0842 | 14.6321 | 449.18 | 0.3239 | 1.2976 |
| 1 | 2.9692 | 0.6467 | 0.0526 | 0.1039 | 17.5951 | 314.62 | 4.7123 | 16.5402 |
| 3 | 2.3169 | 2.5050 | 0.0310 | 0.1134 | 57.6529 | 272.11 | 117.4 | 38.0012 |
| 5 | 1.3142 | 1.4341 | 0.0152 | 0.0480 | 10.6637 | 20.22 | 101.1 | 0.6900 |
| 10 | 0.5148 | 0.8977 | 0.0175 | 0.0783 | 10.1016 | 16.042 | 131.42 | 112.52 |

**Table S1. 13**: Units for parameters in Table S1. 12.

|  | $\boldsymbol{\alpha}_{\boldsymbol{m}_{\boldsymbol{1}}}$ | $\boldsymbol{\beta}_{\boldsymbol{m}_{\boldsymbol{1}}}$ | $\boldsymbol{\alpha}_{\boldsymbol{m}_{\boldsymbol{2}}}$ | $\boldsymbol{\beta}_{\boldsymbol{m}_{\boldsymbol{2}}}$ | $\boldsymbol{\alpha}_{\boldsymbol{h}_{\boldsymbol{1}}}$ | $\boldsymbol{\beta}_{\boldsymbol{h}_{\boldsymbol{1}}}$ | $\boldsymbol{\alpha}_{\boldsymbol{h}_{\boldsymbol{2}}}$ | $\boldsymbol{\beta}_{\boldsymbol{h}_{\boldsymbol{2}}}$ |
| --- | --- | --- | --- | --- | --- | --- | --- | --- |
| Unit | mM^-1^s^-1^ | s^-1^ | s^-1^ | s^-1^ | s^-1^ | s^-1^ | s^-1^ | s^-1^ |

| 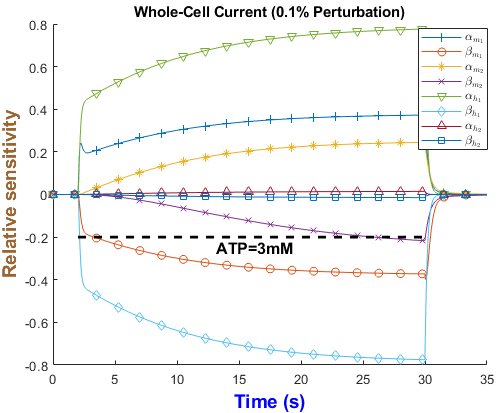  **(A)** | 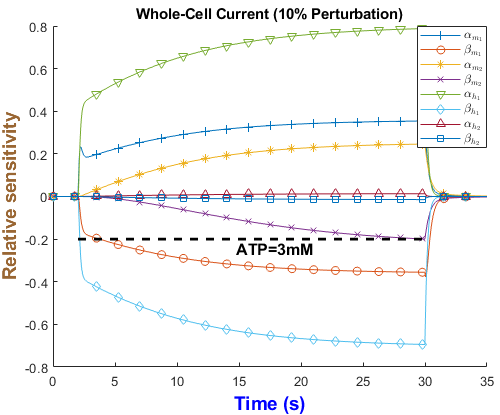  **(B)** |
| --- | --- |

**Figure S1. 6**: Sensitivity analysis of the whole-cell current for ATP=3mM with respect to the parameter set of hP2X_7_ receptor.

**Human GluA1 model**

This section presents the refinement of kinetic parameters for the hGluA1 receptor model across varying levels of Glu, detailed in Tables S1. 14 and S1. 15. Fig. S1. 7 shows the model SA.

**Table S1. 14**: This table displays the rate constants for both activation and deactivation processes of the hGluA1 receptors at different Glu concentrations.

| Glu (mM) | $\boldsymbol{\alpha}_{\boldsymbol{m}_{\boldsymbol{1}}}$ | $\boldsymbol{\beta}_{\boldsymbol{m}_{\boldsymbol{1}}}$ | $\boldsymbol{\alpha}_{\boldsymbol{m}_{\boldsymbol{2}}}$ | $\boldsymbol{\beta}_{\boldsymbol{m}_{\boldsymbol{2}}}$ | $\boldsymbol{\alpha}_{\boldsymbol{h}_{\boldsymbol{1}}}$ | $\boldsymbol{\beta}_{\boldsymbol{h}_{\boldsymbol{1}}}$ | $\boldsymbol{\alpha}_{\boldsymbol{h}_{\boldsymbol{2}}}$ | $\boldsymbol{\beta}_{\boldsymbol{h}_{\boldsymbol{2}}}$ |
| --- | --- | --- | --- | --- | --- | --- | --- | --- |
| 0.1 | 5.7234 | 0.1436 | 241.42 | 4.1676 | 3.8585 | 15.984 | 0.0168 | 0.15950 |
| 1 | 2.0155 | 0.1160 | 13.092 | 1.3504 | 5.8913 | 3.6551 | 0.0384 | 0.2143 |
| 10 | 0.2908 | 0.2801 | 27.995 | 0.2717 | 2.9469 | 0.8673 | 0.0550 | 0.2053 |

**Table S1. 15**: Units for parameters in Table S1. 14.

|  | $\boldsymbol{\alpha}_{\boldsymbol{m}_{\boldsymbol{1}}}$ | $\boldsymbol{\beta}_{\boldsymbol{m}_{\boldsymbol{1}}}$ | $\boldsymbol{\alpha}_{\boldsymbol{m}_{\boldsymbol{2}}}$ | $\boldsymbol{\beta}_{\boldsymbol{m}_{\boldsymbol{2}}}$ | $\boldsymbol{\alpha}_{\boldsymbol{h}_{\boldsymbol{1}}}$ | $\boldsymbol{\beta}_{\boldsymbol{h}_{\boldsymbol{1}}}$ | $\boldsymbol{\alpha}_{\boldsymbol{h}_{\boldsymbol{2}}}$ | $\boldsymbol{\beta}_{\boldsymbol{h}_{\boldsymbol{2}}}$ |
| --- | --- | --- | --- | --- | --- | --- | --- | --- |
| Unit | mM^-1^ms^-1^ | mM^-1^ms^-1^ | ms^-1^ | mM^-1^ms^-1^ | mM^-1^ms^-1^ | ms^-1^ | mM^-1^ms^-1^ | ms^-1^ |

| 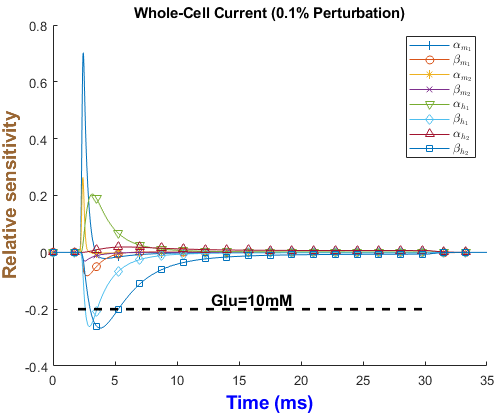  **(A)** | 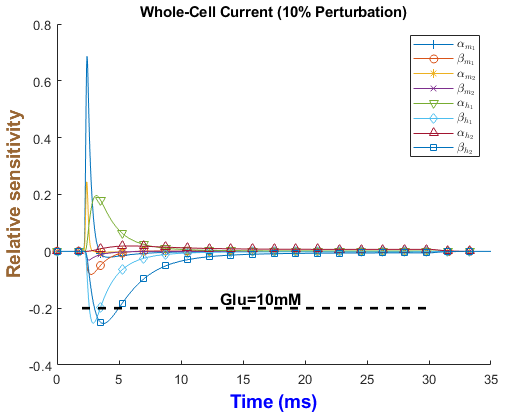  **(B)** |
| --- | --- |

**Figure S1. 7**: Sensitivity analysis of the whole-cell current for Glu=10mM with respect to the parameter set of hGluA1 receptor.

# S1. 2. Pseudo-Reversible (Feedback-based) gHH Variant

The forward-dominant gHH model treats the α and β rate pairs phenomenologically; each gate has a backward flux, but that flux is independent of the companion inactivation variable. To create an explicit reciprocal coupling—without introducing extra states—we multiply every backward rate of the activation gates by its cognate inactivation variable, yielding Eqs S1.1–S1.2.
This modification restores a bidirectional link at the ODE level but does not by itself impose strict detailed balance (the product constraints on multi-gate cycles). We therefore refer to it as the *h-feedback* or *pseudo-reversible* variant. Fits and statistics for the two formulations are compared below (Table S1.16, Figs. S1.8–S1.10).

| $\frac{\boldsymbol{d}\boldsymbol{m}_{\boldsymbol{1}}}{\boldsymbol{dt}}\boldsymbol{=}\boldsymbol{\alpha}_{\boldsymbol{m}_{\boldsymbol{1}}}\boldsymbol{(A)\times A\times}\left( \boldsymbol{1-}\boldsymbol{m}_{\boldsymbol{1}} \right)\boldsymbol{-}\boldsymbol{\beta}_{\boldsymbol{m}_{\boldsymbol{1}}}\boldsymbol{(A)\times}\left[ \boldsymbol{1+\varphi(A)} \right]\boldsymbol{\times}\boldsymbol{m}_{\boldsymbol{1}}\boldsymbol{\times}\boldsymbol{h}_{\boldsymbol{1}}$ | (Eq. S1.1) |
| --- | --- |
| $\frac{\boldsymbol{d}\boldsymbol{m}_{\boldsymbol{2}}}{\boldsymbol{dt}}\boldsymbol{=}\boldsymbol{\alpha}_{\boldsymbol{m}_{\boldsymbol{2}}}\boldsymbol{(A)\times}{\boldsymbol{m}_{\boldsymbol{1}}}^{\boldsymbol{n}_{\boldsymbol{1}}}\boldsymbol{\times}\left( \boldsymbol{1-}\boldsymbol{m}_{\boldsymbol{2}} \right)\boldsymbol{-}\boldsymbol{\beta}_{\boldsymbol{m}_{\boldsymbol{2}}}\boldsymbol{(A)\times}\left[ \boldsymbol{1+\varphi(A)} \right]\boldsymbol{\times}\boldsymbol{m}_{\boldsymbol{2}}\boldsymbol{\times}\boldsymbol{h}_{\boldsymbol{2}}$ | (Eq. S1.2) |

**Table S1. 16**: Comparison of the sum-of-squared-errors (SSE) obtained with the forward-only gHH model versus the reversible h-feedback variant (Eqs S1.1–S1.2). ΔSSE is expressed relative to the forward-only value; positive numbers indicate a worse fit. The h-feedback modification therefore offers no statistical advantage for any of the three receptors.

| Receptor | Agonist | SSE forward-only | SSE + h-feedback | ΔSSE | Preferred |
| --- | --- | --- | --- | --- | --- |
| hP2X_4_ | ATP 50 µM | 0.7133 | 0.9332 | +24% | forward |
| hP2X_7_ | ATP 3 mM | 5.412 ×10⁻⁴ | 8.039 ×10⁻⁴ | +49% | forward |
| hGluA1 | Glu 10 mM | 4.6334 | 4.6820 | +1% | forward |

| 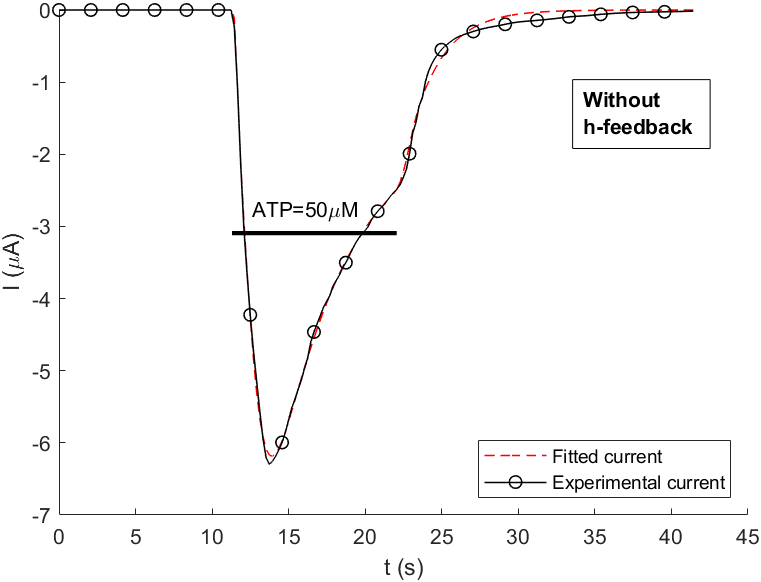  **(A)** | 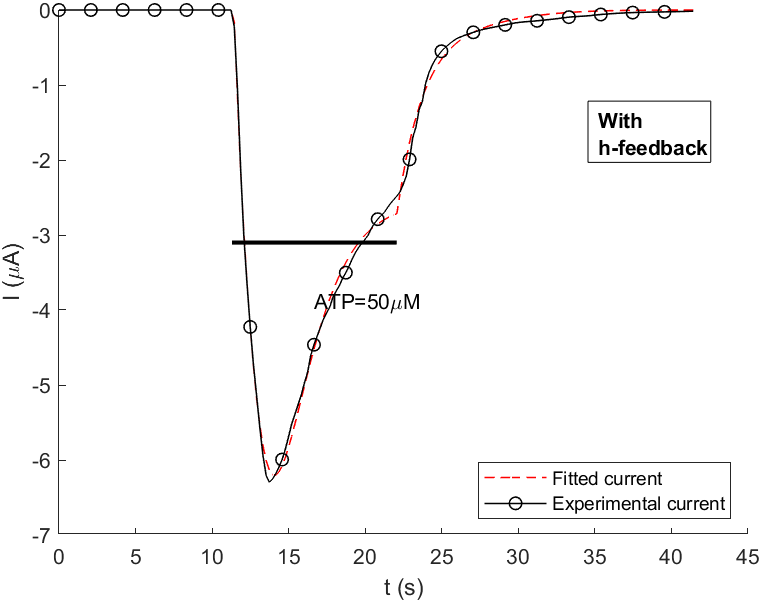  **(B)** |
| --- | --- |

**Figure S1. 8**: Fit quality for hP2X_4_ at 50 µM ATP. (A) Forward-only gHH model (main-text equation): SSE = 0.7133. (B) Reversible h-feedback variant (Eqs S1.1–S1.2): SSE = 0.9332 (ΔSSE = +2 %). The reversible scheme produces a slightly slower recovery phase and larger residuals, confirming that the forward-dominant formulation already captures the macroscopic kinetics.

| 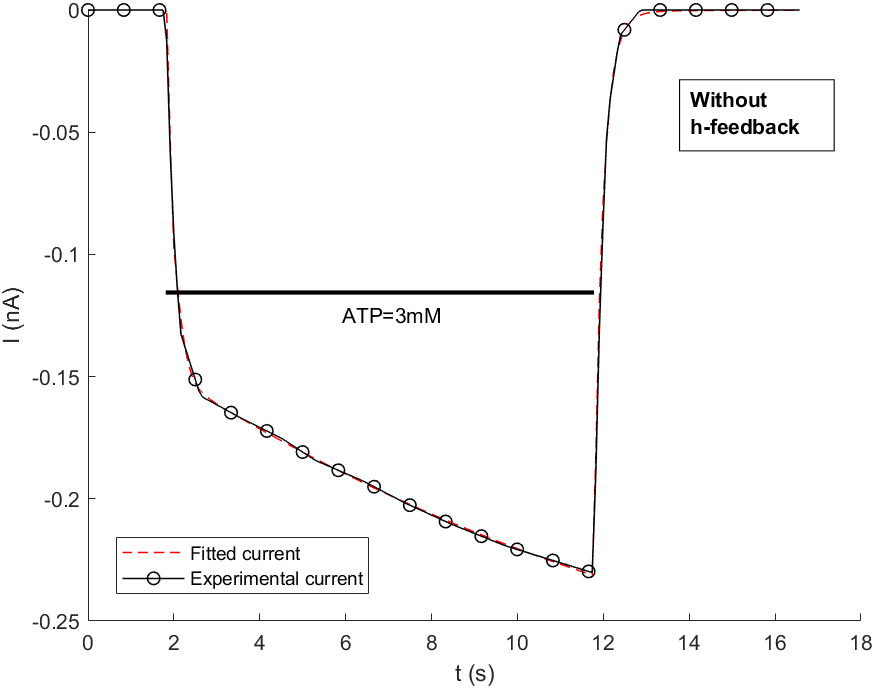  **(A)** | 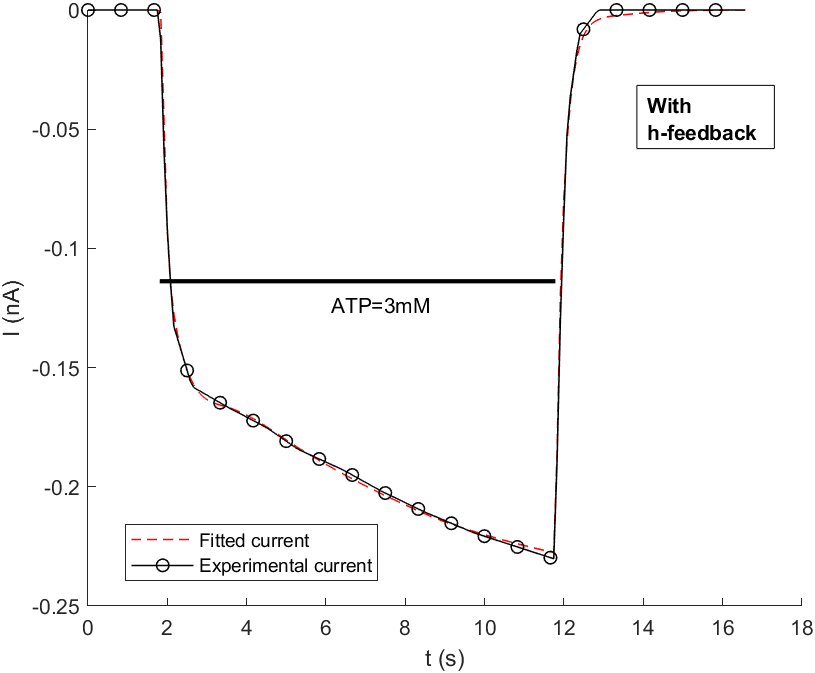  **(B)** |
| --- | --- |

**Figure S1. 9**: Fit quality for hP2X_7_ at 3 mM ATP. (A) Forward-only gHH model: SSE = 5.412 × 10⁻⁴. (B) Reversible h-feedback variant: SSE = 8.039 × 10⁻⁴ (ΔSSE = +49%). Even for this slowly desensitising isoform, enforcing explicit feedback degrades the fit, indicating that microscopic, detailed balance is not required to reproduce whole-cell currents.

| 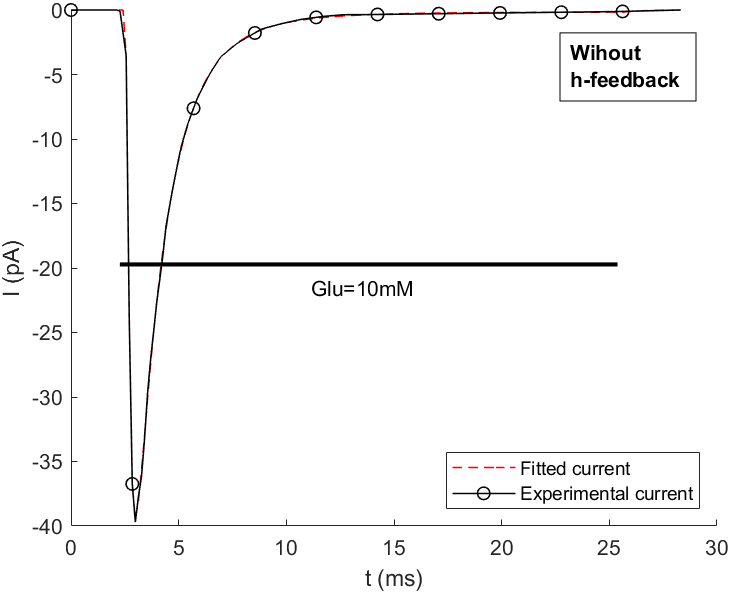  **(A)** | 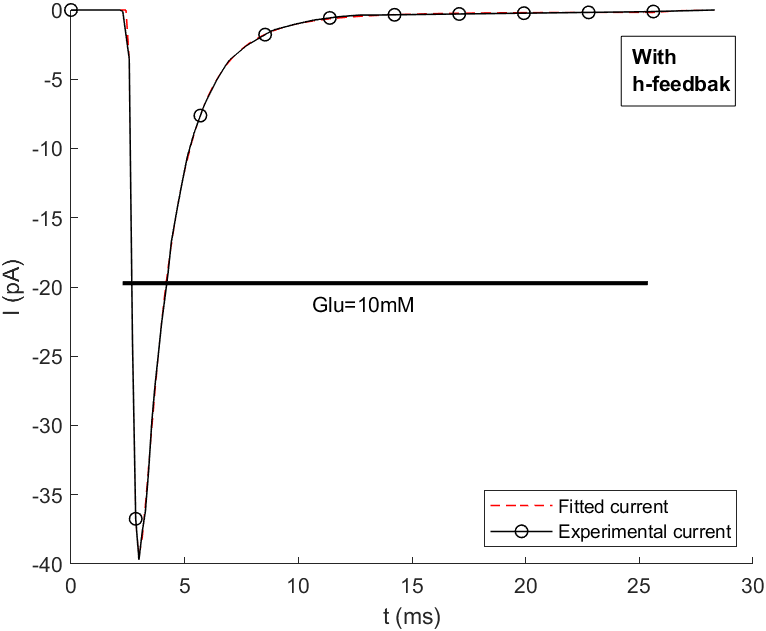  **(B)** |
| --- | --- |

**Figure S1. 10**: Fit quality for hGluA1 at 10 mM glutamate. (A) Forward-only gHH model: SSE = 4.6334. (B) Reversible h-feedback variant: SSE = 4.6820 (ΔSSE = +1%). The two models are visually indistinguishable; the minimal SSE increase further supports the choice of the simpler forward-dominant form for macroscopic analyses.

# S1. 3. Contribution of Model Components

We rebuilt the gHH model with only one activation ($m_{1}$) and one inactivation ($h_{1}$) gate, refitted the reduced system, and compared it with the full four-gate model. Figure S1.11 shows four representative receptors (hP2X_1_, hP2X_4_, hP2X_5_, hP2X_7_). In every case the two-gate variant could not replicate the rising phase and/or late recovery, confirming that m₂ and h₂ are essential for a unified description across receptors. These experiments demonstrate that the four-gate scaffold is the minimal model that yields consistent, high-quality fits for all eight human receptors over 38 current recordings.

| 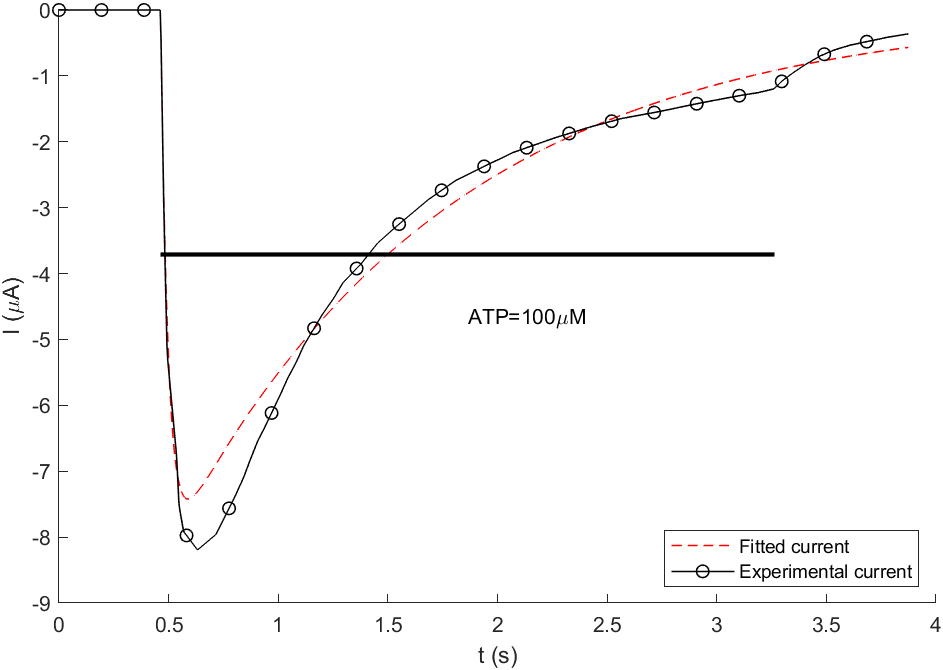  **(a)** | 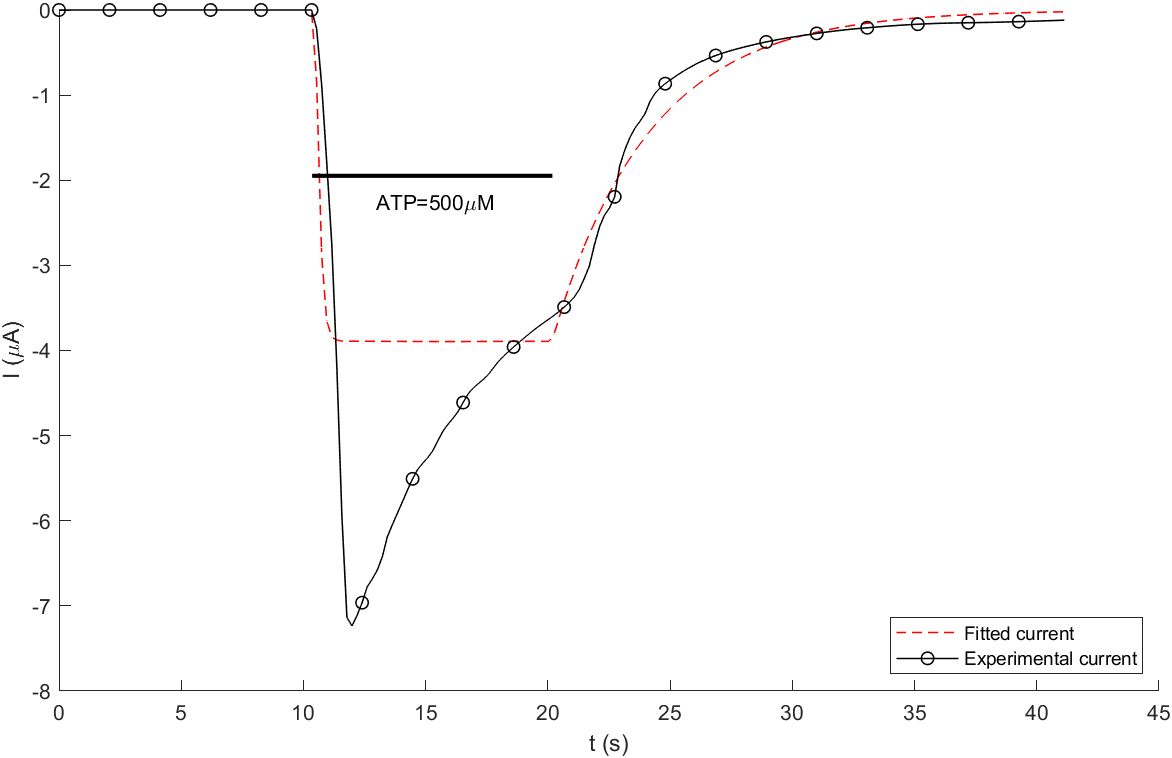  **(b)** |
| --- | --- |
| 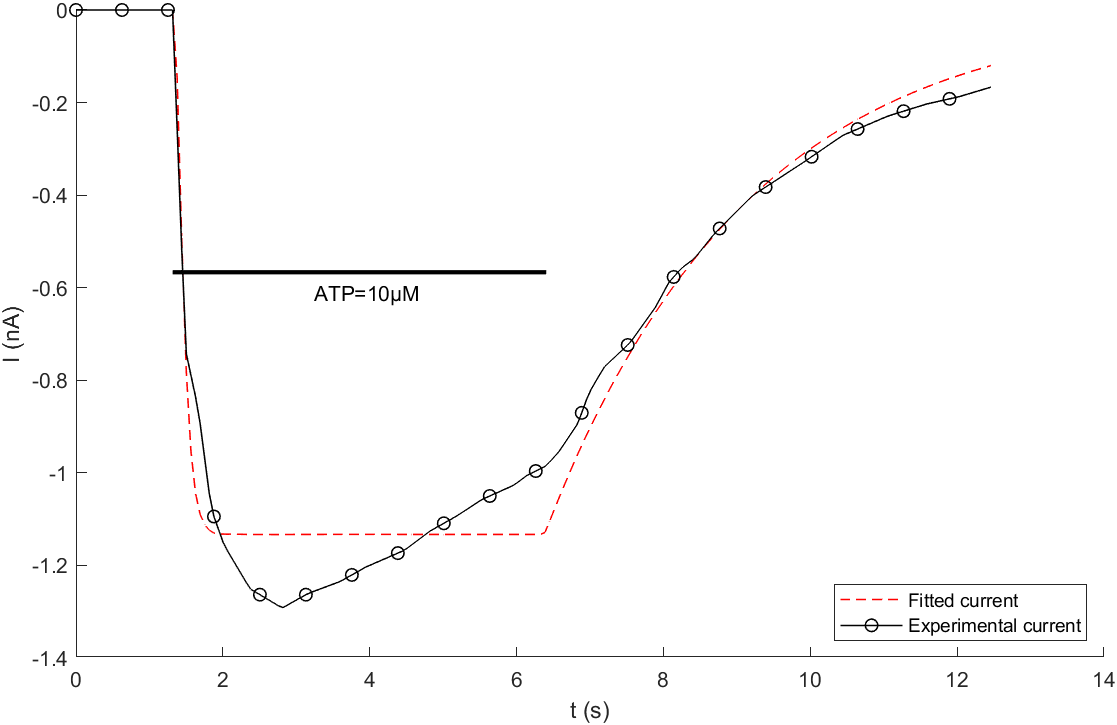  **(c)** | 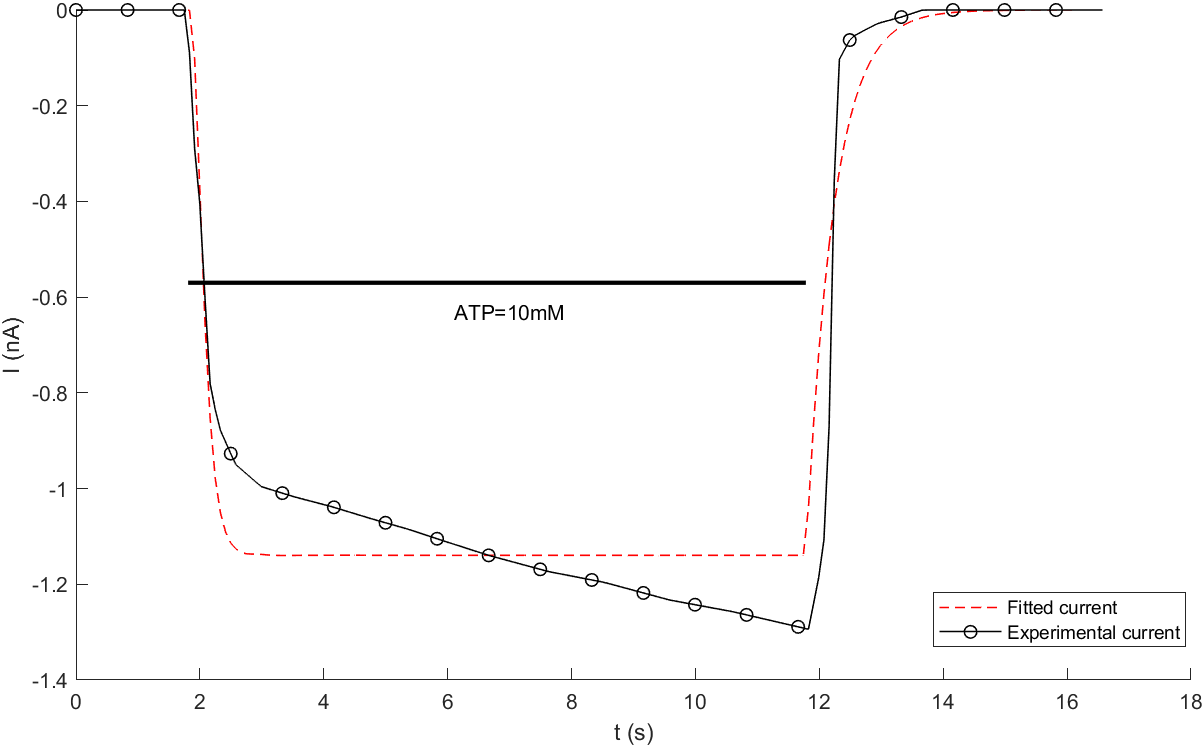  **(d)** |

**Figure S1. 11**: Failure of the two-gate variant. Dashed red = best fit obtained with only m₁ and h₁ gates; solid black = experimental trace. Panels: (a) hP2X_1_ (ATP 100 µM); (b) hP2X_4_ (ATP 500 µM); (c) hP2X_5_ (ATP 10 µM); (d) hP2X_7_ (ATP 10 mM). The reduced model consistently underestimates the initial overshoot and/or the slow recovery, whereas the full four-gate model (see main figures) matches all epochs, underscoring the need for the complete $m_{1}-m_{2}-h_{1}-h_{2}$ scheme.

# S1. 4. Recovery Time of hP2X_1_ and hP2X_2_ Receptors

To benchmark the seconds-scale recovery of hP2X_1-2_ against the minute-scale recovery of hP2X₃, we simulated 2-s ATP pulses over the full concentration range and measured the time for full recovery. Results (Fig. S1.12) show that both hP2X₁ and hP2X₂ recovers within seconds [1], while prolonged recovery is unique to hP2X₃ [2].

| 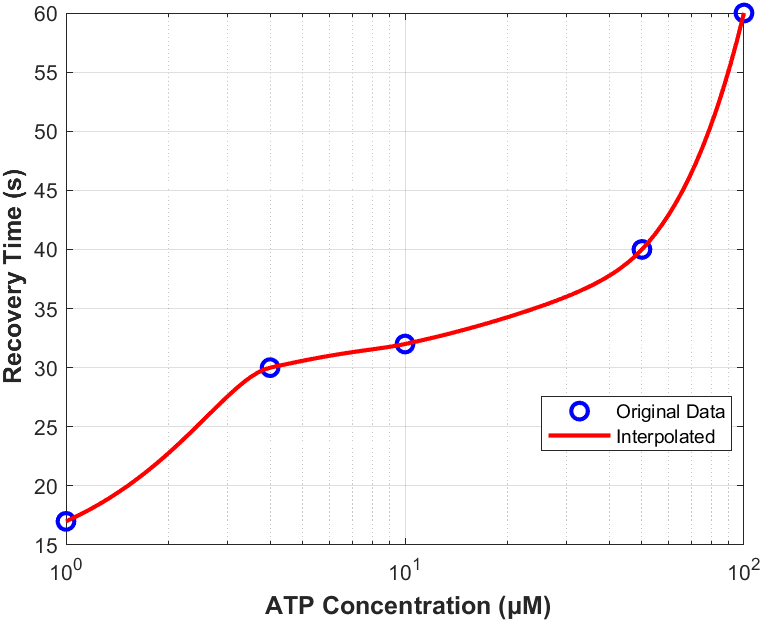  **(A)** | 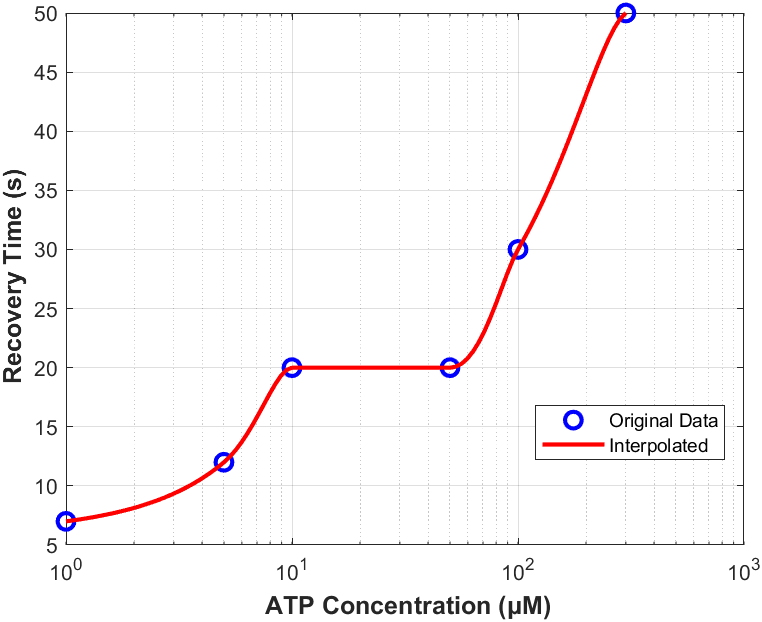  **(B)** |
| --- | --- |

**Figure S1. 12**: **Full recovery time of hP2X_1_ and hP2X_2_ Receptors.** (A) hP2X_1_ (B) hP2X_2_. The x-axis is logarithmic. These seconds-scale values contrast with the minute-scale recovery of hP2X_3_ (Fig. 13).

# S1. 5. References

[1] Cook S, Rodland K, McCleskey E. A memory for extracellular Ca2+ by speeding recovery of P2X receptors from desensitization. J Neurosci 1998;18:9238–44.

[2] Pratt EB, Brink TS, Bergson P, Voigt MM, Cook SP. Use-dependent inhibition of P2X3 receptors by nanomolar agonist. J Neurosci 2005;25:7359–65.

# S2. Data

All digitised datasets extracted from human experiments referenced in this article are fully described and documented at <https://github.com/poshtkohi/gHH/tree/main/hP2XR-hGluAR-model/data>. The public repository includes a README file that specifies the units and receptor subtypes with the relevant experimental conditions.

# S3. Code

All MATLAB source code of the gHH model, including parameter fitting and simulations, can be found on the GitHub page at <https://github.com/poshtkohi/gHH>. It comes with a README file that explains the source code hierarchy.
